# An AI-driven hybrid random forest-LSTM framework for IoT-based groundwater level prediction and sustainable water management in Chennai, India

**DOI:** 10.3389/frai.2026.1958933

**Published:** 2026-10-01

**Authors:** S. Boomika, R. Sitharthan, R. Tanukasree, R. Senthil Kumar

**Affiliations:** 1 School of Electrical Engineering, Vellore Institute of Technology, Chennai, India; 2 Centre for Smart Grid Technologies, Vellore Institute of Technology, Chennai, India

**Keywords:** aquifer system, artificial intelligence, groundwater level prediction, internet of things (IoT), machine learning, sustainable water management

## Abstract

**Introduction:**

Groundwater resources in Chennai, Tamil Nadu, India are under increased stress due to rapid urbanization, erratic monsoon patterns and largely unregulated groundwater extraction. Addressing this challenge requires not only continuous monitoring but also accurate forecasting of groundwater fluctuations.

**Methods:**

This study presents a data driven framework that combines Internet of Things (IoT) enabled sensing with Machine Learning model. Real-time measurements such as groundwater depth, rainfall, temperature, and soil moisture are combined with historical hydro-meteorological data to build a reliable prediction system. A hybrid modelling approach is adopted in this study, where Random Forest is used to identify key influencing factors and capture non-linear relationships, while Long Short-Term Memory (LSTM) networks are employed to model temporal variations.

**Results:**

The combined RF-LSTM model surpasses traditional techniques, delivering an RMSE of 0.38 m, an MAE of 0.29 m, and R^2^ value of 0.96. Relative to a standalone LSTM model, the hybrid approach cuts the RMSE by approximately 22%, and it also outperforms Random Forest, Support Vector Regression, and Linear Regression individually.

**Discussion:**

The model successfully tracks seasonal shifts in groundwater capturing groundwater recharge during the Northeast monsoon and depletion during the summer season. Through timely and dependable forecasts, this system aids more effective groundwater management, strengthens rainwater harvesting efforts and supports sustainable urban water practices going forward.

## 1 Introduction

Groundwater serves as a vital resource for satisfying the water needs of fast expanding urban areas, especially in cities where surface water availability is scarce or inconsistent. Chennai, the capital of Tamil Nadu, illustrates this reliance clearly. During the last twenty years, the city has undergone substantial population growth, industrial development, and urban expansion, collectively straining its groundwater reserves (Muthu and Thirumalaisamy, 2025). Simultaneously, shifting rainfall trends, particularly the inconsistency of the Northeast monsoon has rendered natural recharge harder to predict (Venkatesan and Sinduja, 2026). Consequently, groundwater levels across several parts of the city have dropped, with certain areas facing severe scarcity during dry periods.

Historically, groundwater monitoring in Chennai has depended on periodic manual readings taken from observation wells. Physically based numerical models such as MODFLOW, simulate groundwater movement through governing differential equations and demand detailed aquifer and hydraulic property inputs for precise results (Sulis et al., 2010). The water table fluctuation technique estimates recharge based on rises in groundwater levels, though it offers only intermittent insights and fails to capture the continuous changes necessary for real time decisions (Yang et al., 2018). Additionally, field monitoring depends on extended data collection from observation wells, which supports trend analysis critical to understanding and managing aquifer depletion (Du et al., 2024). Time series analysis draws on historical groundwater and climatic records to project future water levels by identifying connections among variables like rainfall and temperature (Patle et al., 2015). Conventional groundwater monitoring approaches tend to be data-heavy, time consuming, and expensive due to the scale of field measurements and modelling work involved, and their spatial coverage is constrained since point-based observations cannot fully represent large aquifer systems (Hanke, 1970; Masood et al., 2022). More importantly, these methods lack robust forecasting ability, since they mainly describe historical conditions rather than predicting future groundwater trends. In contrast, traditional approaches are manual, labor-intensive, limited in spatial and temporal resolution, and poorly suited for predictive analysis.

Recent research has increasingly demonstrated the strength of Machine Learning methods for predicting groundwater levels relative to conventional statistical techniques. ML-driven approaches have delivered marked gains in prediction accuracy by capturing intricate patterns directly from historical records. A comprehensive review and meta-analysis covering 197 studies is presented in (Ahmadi et al., 2022), which finds that machine learning models are particularly effective for groundwater level forecasting, with accuracy depending more on input data quality than on the specific algorithm chosen. This review also confirms that data-driven techniques, particularly neural networks, substantially improve the accuracy and efficiency of groundwater monitoring and prediction. Groundwater level forecasting through machine learning in a drought affected region is examined in (Pham et al., 2022), where ensemble methods, notably Bagging RT and Bagging RF, are shown to yield the highest prediction accuracy with minimal error and strong correlation, positioning them as valuable tools for sustainable groundwater management. A systematic review of ML techniques applied to groundwater level forecasting appears in (Jesse et al., 2025), noting that ANN, SVM, LSTM and RF are commonly used, and systematic tuning hinders the creation of fully reliable forecasting systems.

Likewise, deep learning approaches particularly Long Short-Term Memory (LSTM) networks have drawn considerable interest in forecasting time series hydrological data. As noted in (Wunsch et al., 2021; Seo and Lee, 2021), LSTM based models excel at capturing long range temporal dependencies within groundwater fluctuations particularly in areas marked by pronounced seasonal change. These models can learn the delayed relationship between rainfall events and groundwater recharge, a nuance often overlooked by conventional techniques.

In recent times, incorporating Internet of Things technology into hydrological monitoring has substantially reshaped how groundwater resources are tracked and studied (Miller et al., 2025). IoT facilitates continuous, real time data collection from distributed sensor networks positioned in borewells and recharge areas, recording essential parameters including groundwater level, rainfall, temperature and soil moisture. This transition from intermittent manual readings to automated sensing systems delivers far greater temporal resolution, which is vital for grasping dynamic groundwater behaviour within fast evolving urban settings. Pairing IoT with data-driven analytics also unlocks new ways for predictive modelling. Rather than depending exclusively on historical data, real time sensor readings can be fed directly into machine learning models to enhance both forecasting accuracy and responsiveness (Banerjee et al., 2026; Prabu et al., 2024). This holds particular significance in areas where groundwater levels shift quickly due to seasonal monsoon variability and changing extraction rates. Through the integration of IoT infrastructure with sophisticated machine learning methods, it becomes feasible not only to track present groundwater conditions but also to forecast future patterns with enhanced confidence.

The central contribution of this study is the creation of a fully integrated, real time groundwater intelligence framework that merges IoT based sensing infrastructure with a hybrid machine learning architecture to enable high resolution prediction of groundwater dynamics within complex urban aquifer systems. Unlike much of existing literature, which largely depends on either fixed historical datasets or standalone predictive models with limited capacity for temporal adaptation, the proposed approach builds a continuous data to decision pipeline that directly integrates real time hydrological signals with historical hydro meteorological trends. The novelty of this work is threefold. First, it introduces a real-time IoT-driven monitoring layer that captures high-frequency groundwater fluctuations, enabling dynamic system updates rather than retrospective analysis. Second, it proposes a hybrid learning strategy that synergistically integrates Random Forest-based feature intelligence with Long Short-Term Memory (LSTM) networks, allowing simultaneous modelling of non-linear environmental interactions and long-term temporal dependencies. Third, and most critically, the framework explicitly embeds urban-specific hydrogeological drivers such as heterogeneous extraction intensity, rapid land-use transformation, and decentralized artificial recharge mechanisms, thereby overcoming the key limitation of generalized models that ignore local system heterogeneity. The study further evaluates the hybrid model against standalone and conventional machine-learning baselines and examines its suitability for real-time groundwater decision support. Table 1 summarizes the distinction between the present framework and representative groundwater and hydrological machine-learning approaches reported in the literature. Overall, the proposed framework is highly useful for society as it supports informed water resource planning, reduces the risk of groundwater depletion, and directly contributes to achieving key Sustainable Development Goals, particularly SDG 6 (Clean Water and Sanitation), by promoting efficient, equitable, and long-term sustainable groundwater management.

**Table 1:** Comparison of the proposed approach with representative groundwater prediction approaches.

Study/Approach	Main modelling approach	Data/Inputs	Temporal/real-time capability	Distinction from present study
Physics-based numerical models (Sulis et al., 2010)	Governing equations and aquifer parameters	Hydraulic properties, boundary conditions, recharge and extraction data	Can represent dynamic processes but requires detailed parameterization	Provides mechanistic representation but requires extensive aquifer characterization
Water-table fluctuation method (Yang et al., 2018)	Water-level rise/fall based recharge estimation	Observation-well groundwater levels and recharge information	Generally observation-dependent and intermittent	Useful for recharge estimation but does not provide a hybrid ML forecasting architecture
LSTM-based groundwater prediction (Wunsch et al., 2021; Seo and Lee, 2021)	Long Short-Term Memory neural network	Historical groundwater and hydro-meteorological time series	Strong temporal learning capability	Does not explicitly employ RF-based feature-importance analysis before temporal modelling
RF-based groundwater prediction (Pham et al., 2022; Jesse et al., 2025)	Random Forest/ensemble learning	Groundwater and hydro-meteorological variables	Captures nonlinear relationships but has limited intrinsic sequence memory	The proposed framework uses RF for feature intelligence followed by LSTM for temporal dependency modelling
IoT + Machine Learning (Banerjee et al., 2026; Prabu et al., 2024)	IoT sensing combined with ML models	Real-time sensor and/or historical environmental observations	Supports continuous monitoring	The present study integrates IoT sensing with RF feature selection and LSTM sequence forecasting
Proposed	RF feature selection + two-layer LSTM	Groundwater level, rainfall, temperature, soil moisture, humidity and extraction-related features	Continuous sensing with sequential forecasting	Integrates IoT sensing, RF-based feature intelligence and LSTM temporal learning for localized urban groundwater prediction in Chennai

This paper is organized as follows. Section 2 describes the system architecture of the proposed system. Section 3 discusses data collection and preprocessing. Section 4 discusses the architecture of the proposed hybrid RF-LSTM model. Section 5 discusses results and evaluation of the proposed model. Finally, Section 6 concludes the paper and outlines future research directions.

## 2 System architecture

Figure 1 depicts a layered architecture for a smart groundwater monitoring and prediction system, where each stage is closely interconnected to support continuous sensing, dependable data transmission, and intelligent decision making. At the front end, the sensing layer comprises distributed IoT nodes positioned in borewells and adjacent soil areas. Groundwater level is measured using either ultrasonic or pressure-based sensors, chosen according to installation depth and environmental conditions. Ultrasonic sensors determine water level through time-of-flight measurements, while pressure sensors deduce depth from hydrostatic pressure readings. Supplementary environmental factors like soil moisture, ambient temperature, and rainfall are gathered via dedicated probes. These varied sensors produce time stamped, multi-dimensional data reflecting both subsurface and surface hydrological conditions. The signals connect to an IoT edge device or gateway, which handles preliminary functions such as sensor polling, analog to digital conversion, basic filtering, and packet formation.

**Figure 1:**
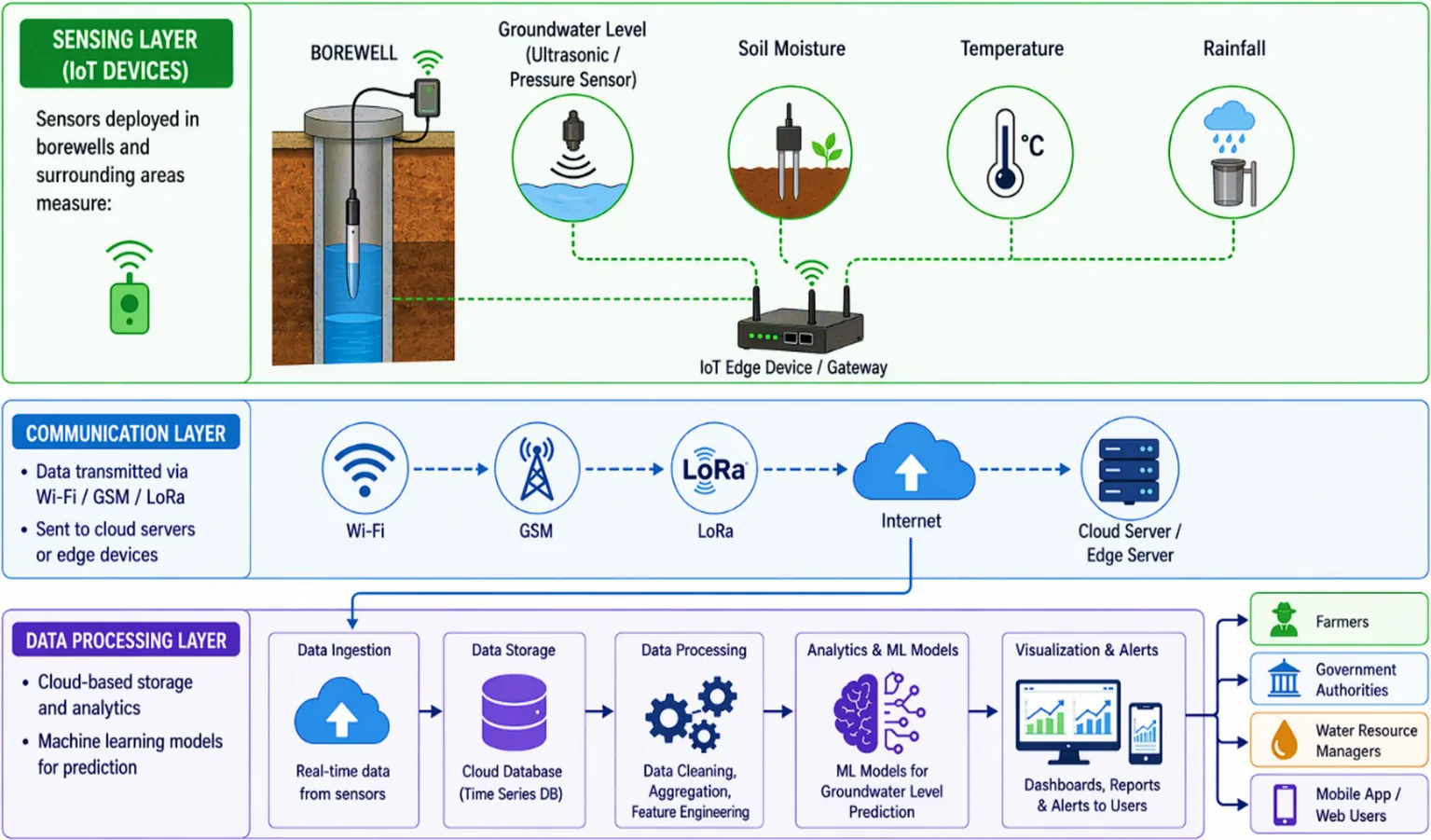
Architecture of smart groundwater monitoring and prediction system.

The communication layer guarantees that field level data reaches higher level computing infrastructure with minimal delay and loss. Depending on deployment factors like range, power supply, and network availability, the system accommodates multiple communication protocols, including Wi-Fi, GSM, LoRa. Wi-Fi suits short range, high bandwidth applications, GSM offers broad connectivity via cellular networks, and LoRa supports low power, long distance transmission in rural or remote settings. The gateway dynamically handles protocol selection and data routing, encapsulating sensor data into structured payloads and forwarding them via the internet to cloud or edge servers. This layer plays a critical role in maintaining data integrity, synchronization, and secure transmission.

Once the data arrives at the processing layer, it goes through a series of conceptual steps intended to convert raw measurements into actionable insights. Data ingestion modules first manage real time streaming inputs, organizing them within cloud-based storage systems, typically time series databases designed for continuous sensor data. Preprocessing stages then handle noise removal, missing value treatment, normalization, and feature extraction to enhance data quality. The refined dataset is then passed into machine learning models trained to identify temporal patterns and relationships among variables like rainfall, soil moisture and groundwater levels. These models support predictive analytics particularly in forecasting groundwater fluctuations and flagging potential water scarcity situations.

The system’s final stage centers on visualizing and sharing results. Analytical findings are delivered through dashboards, reports and automated alert systems, offering both real time monitoring and future projections. Alerts may be triggered by pre-defined thresholds or model outputs, such as signs of rapid groundwater depletion or drought risk. This information reaches various stakeholders including farmers, government bodies and water resource managers via web and mobile platforms.

## 3 Data collection and preprocessing

Figure 2 represents the groundwater level datasets utilized in this study. Figure 2A displays a nationwide depth to water level distribution map of India for 2026, sourced from the Central Ground Water Board (CGWB). This map represents groundwater depth in meters below ground level (m bgl), using color gradients that span from shallow zones (0 to 2 m) to severely depleted zones (over 40 m). Gauge station locations marked on the map correspond to the monitoring wells from which groundwater data were gathered. The figure highlights considerable groundwater stress across northwestern India, whereas the southern and northwestern regions exhibit comparatively healthier groundwater conditions. This study focuses specifically on the Tamil Nadu region for detailed analysis.

**Figure 2:**
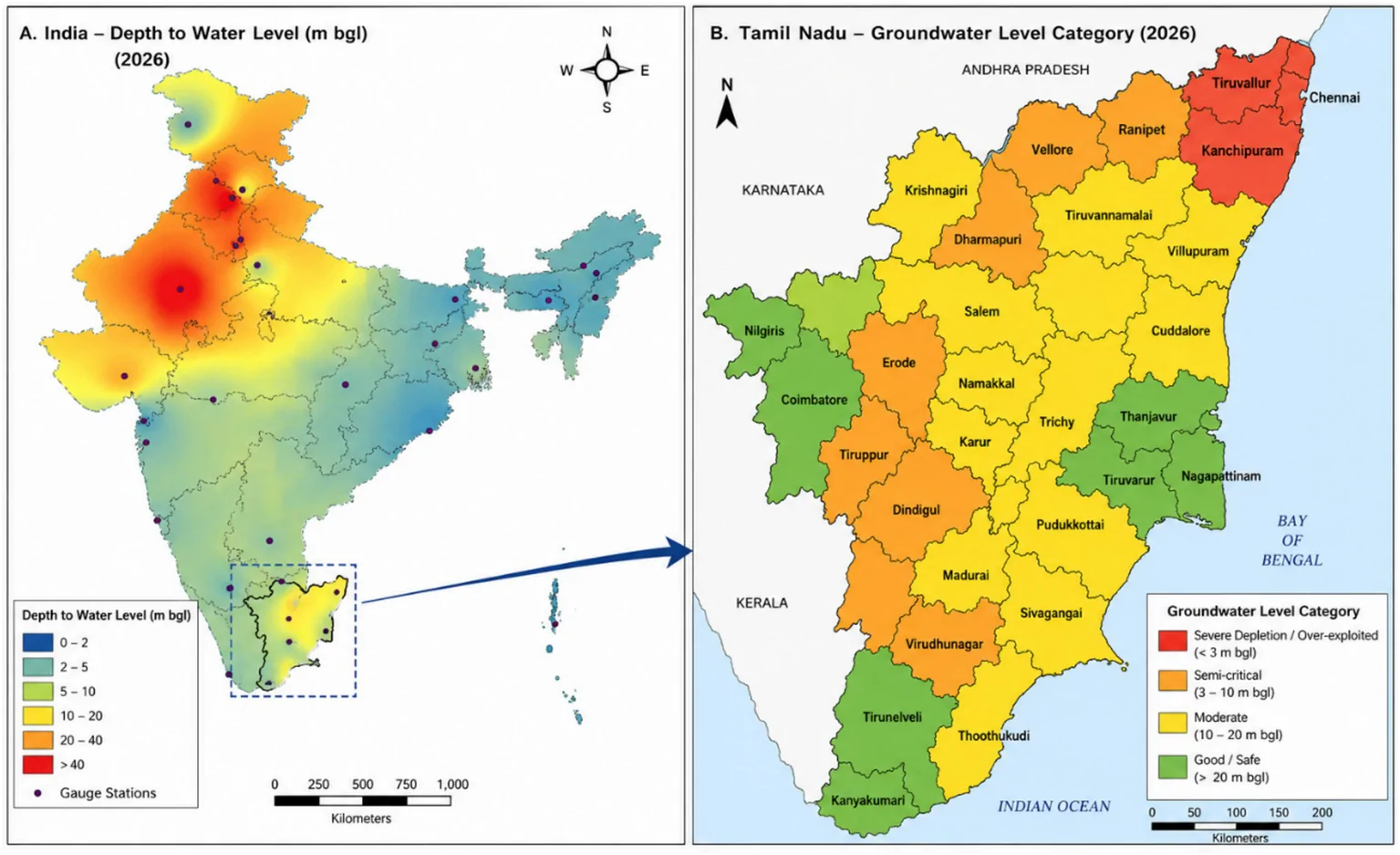
Groundwater level distribution used in this study: **(A)** national groundwater depth distribution and monitoring-station locations; **(B)** enlarged groundwater status map of Tamil Nadu showing severe depletion, semi-critical, moderate, and good/safe zones (source: Central Ground Water Board, India-water level year 2025–2026).

Figure 2B presents an enlarged thematic groundwater map of Tamil Nadu. This state level dataset classifies districts into four groundwater status categories which are severe depletion/over exploited, semi critical, moderate, and good/safe zones. Districts such as Chennai, Tiruvallur and Kanchipuram exhibit severe groundwater depletion, driven by extensive urbanization and extraction, Conversely, districts including Coimbatore, Tirunelveli, Kanyakumari, Thanjavur and Nagapattinam demonstrate more favorable groundwater recharge conditions. The groundwater data used in this work were obtained from CGWB monitoring stations and regional hydrological assessment reports. During preprocessing, spatial interpolation was carried out, missing observations were removed, groundwater depth values were normalized, and data were categorized by district to support visualization and analysis. These processed datasets were subsequently applied to groundwater trend analysis and predictive modelling.

### 3.1 Data sources

The effectiveness of the groundwater level prediction model depends heavily on the quality, consistency and diversity of the datasets gathered. Groundwater behaviour is shaped by numerous environmental and climatic influences; to enhance prediction accuracy, data were sourced from a range of dependable hydrological and meteorological sources. The dataset includes historical groundwater records, real-time IoT sensor readings, and climatic factors like rainfall, temperature, and humidity. Combining these different data sources allows for accurate predictions of groundwater changes under various environmental conditions. In this study, the main groundwater data came from the Central Ground Water Board (CGWB), Government of India. We also used additional datasets from the Tamil Nadu Water Resources Department (WRD) and the Public Works Department (PWD) (Ground Water Level CGWB Tamil Nadu, 2026). The collected data were categorized by district based on groundwater stress conditions, including severe depletion, semi-critical, moderate, and good or safe recharge zones, as shown in Table 2.

**Table 2:** Tamil Nadu groundwater data.

District category	Districts	Groundwater status (2026)	Data source
Severe depletion	Chennai, Tiruvallur, Kanchipuram	< 3 m bgl	CGWB, WRD
Semi-critical	Vellore, Ranipet, Dharmapuri, Erode, Tiruppur, Dindigul, Virudhunagar	3–10 m bgl	CGWB
Moderate	Krishnagiri, Tiruvannamalai, Villupuram, Salem, Namakkal, Karur, Trichy, Pudukkottai, Madurai, Sivagangai, Thoothukudi, Cuddalore	10–20 m bgl	CGWB, PWD
Good/Safe	Coimbatore, Nilgiris, Thanjavur, Tiruvarur, Nagapattinam, Tirunelveli, Kanyakumari	Good Recharge Zone	CGWB, WRD

In this study, we also obtained historical groundwater data and meteorological data from government geospatial databases and environmental databases. The historical data relate to the state of groundwater, which is very significant in analyzing the fluctuations of the groundwater levels in different seasons. Also, meteorological elements such as temperature, humidity, etc., will assist in the prediction of evaporation, infiltration, and soil moisture. Hence, such elements are necessarily considered as variables for the ML training system.

#### 3.1.1 Sensor data (real-time)

This study uses IoT technology, which makes the whole process less dependent on humans, thus increasing efficiency. Figure 3 shows the sensor-based real-time groundwater monitoring system. In this study, the data is generated by placing sensors within bore wells and the surroundings. Ultrasonic sensors and pressure sensors are also used to measure water levels. Ultrasonic sensors are used to measure the level of water because it directly depends on measuring the time taken by the ultrasound wave to travel to the water and bounce back after hitting the water. For measurement, the ultrasonic sensor is installed at the upper section of the bore well and oriented vertically toward the groundwater surface. On the other hand, pressure sensors work on the principle of measuring water based on hydrostatic pressure. Data on soil moisture, soil temperature, humidity, and rainfall are collected using dedicated sensors. Communication protocols, including Wi-Fi, have been used to transmit the real-time data generated from the sensors to the database. The time gap between when the data is generated and when it is transmitted may not be constant. Depending on network conditions, transmission delays may range from a few minutes to several hours. Such data allows one to measure the exhaustion of the water level in the resource, whether through exploitation or due to the monsoon season. Further, when it comes to data storage, the collected data is stored in the cloud server for further analysis.

**Figure 3:**
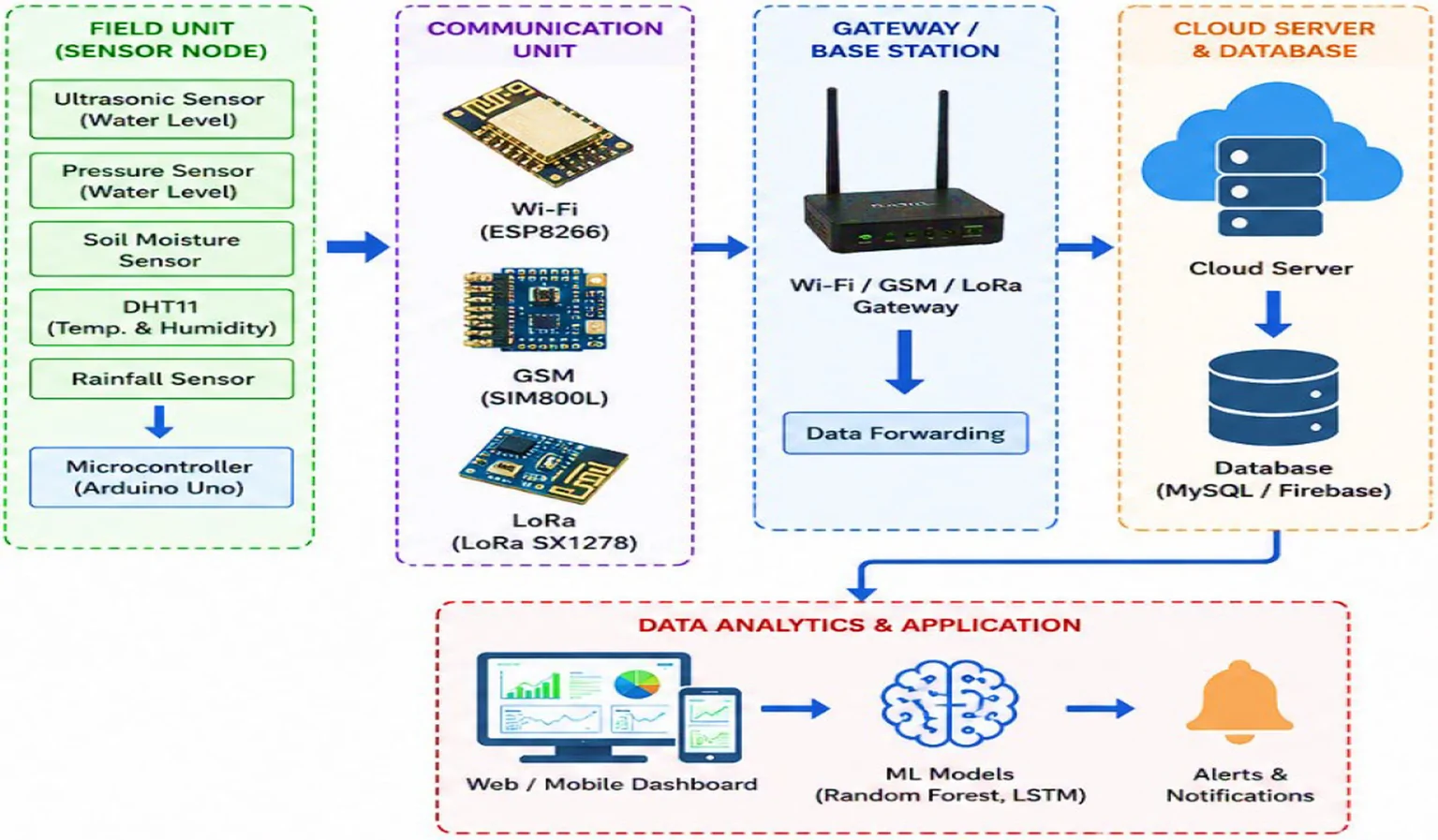
Integrated sensor network for groundwater data acquisition and analytics.

### 3.2 Data preprocessing

Figure 4 shows the data preprocessing pipeline used for predicting groundwater levels. The data collected from various sources may have missing values, noise, inconsistencies, and unnecessary features. Such factors can undermine the performance of machine learning algorithms. Preprocessing is therefore an essential step before deploying prediction models like random forest and LSTM (Mahmud et al., 2026). This stage improves data quality, consistency, reliability, and interpretability, which subsequently enhances the prediction accuracy of the groundwater monitoring system. The pre-processing techniques applied in this study are listed in Table 3. A major challenge in IoT based groundwater monitoring systems is missing data resulting from faulty sensors, communication failures, power outages or incomplete historical records. Missing values can adversely affect model training and lead to inaccurate groundwater predictions. In this study, missing data is addressed through interpolation methods suited to time series groundwater records, along with statistical techniques such as mean and median substitution when needed. Data collected via IoT sensors may also contain noise arising from environmental disturbances, hardware limitations and transmission delays. To address this, noise filtering is performed to improve signal quality and remove unwanted fluctuations in the groundwater data. This work employs moving average filters to smooth out short term variations and low pass filters to eliminate high frequency noise. These methods help preserve genuine variations in groundwater levels while enhancing the reliability of continuous real time monitoring data.

**Figure 4:**
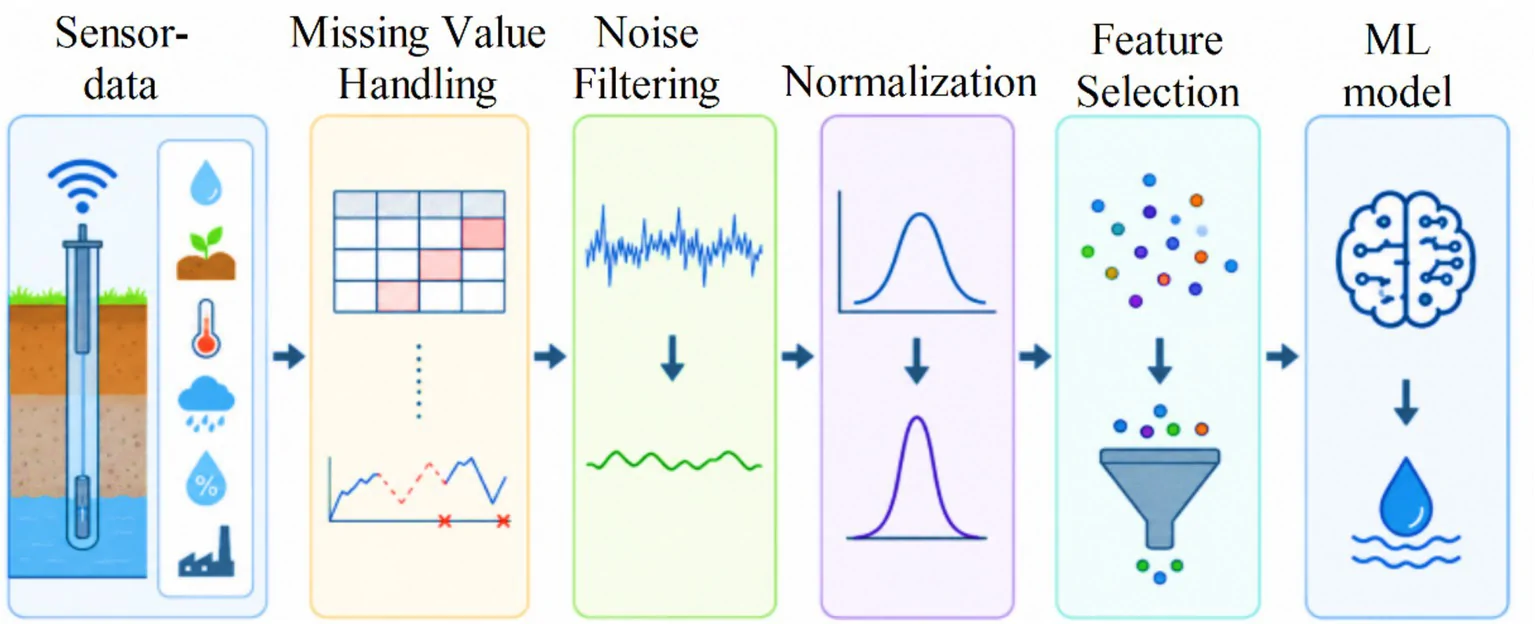
Data preprocessing pipeline for accurate groundwater level prediction.

**Table 3:** Data preprocessing technique considered for accurate groundwater level prediction.

Preprocessing step	Technique used	Purpose
Missing value handling	Interpolation, mean/median filling	Ensure data continuity and avoid incomplete inputs
Noise filtering	Moving average, low-pass filter	Remove fluctuations and improve signal quality
Normalization	Min-max scaling, standardization	Bring all features to a common scale for fair model training
Feature selection	Correlation analysis, statistical tests	Identify important variables and reduce model complexity

Normalization represents another essential preprocessing step. The collected parameters differ in units and value ranges in groundwater level (m), rainfall (mm), temperature (°C) and soil moisture (%) and using such inconsistent ranges directly can reduce the efficiency and stability of machine learning algorithms. To address this, Min-Max normalization and standardization methods are applied to bring all features onto a common numerical scale. Min-Max scaling constrains values between 0 and 1, while standardization adjusts the data distribution to achieve a mean of 0 and variance of 1. This process improves convergence speed and enhances the stability of both Random Forest and LSTM models. The final pre-processing step involves feature selection, aimed at identifying the parameters most influential to groundwater level variations. Since not all collected features contribute equally to prediction accuracy, correlation analysis and statistical significance testing are used to select the most relevant ones. Key parameters identified in this study include rainfall intensity, prior groundwater level, soil moisture, temperature, humidity and water extraction rate. Feature selection reduces redundancy in the dataset, decreases computational complexity, and enhances the interpretability and prediction performance of the proposed framework.

## 4 Proposed hybrid RF-LSTM model

The proposed RF-LSTM hybrid architecture, which merges Random Forest (RF) and Long Short-Term Memory (LSTM) networks for precise groundwater level prediction, is shown in Figure 5. Initially, groundwater and environmental time-series data from IoT sensors and historical records undergo preprocessing. This includes handling missing values, removing noise, and normalizing the data. Next, the RF module evaluates feature importance and selects key hydrological parameters, such as rainfall, previous groundwater level, soil moisture, and temperature. These selected features are then fed into the LSTM network. The network learns long-term dependencies and seasonal variations in groundwater through sequential learning. The extracted temporal features pass through fully connected dense layers to produce predictions of future groundwater levels. The mathematical modelling and function of the proposed RF-LSTM hybrid architecture is given in Equations 1–8 (Mahmud et al., 2026; Asaad et al., 2022; Eryürük and Eryürük, n.d; Boo et al., 2024). The input groundwater feature vector collected from IoT sensors and historical datasets is represented as,

$$X_{t}=[\mathrm{GW}L_{t},R_{t},SM_{t},T_{t},H_{t},ER_{t}]$$ (1)

Where, *GWLT_t_* represents the Groundwater level, *R_t_​* represents the Rainfall, *SM*_*t*​_ represents the soil moisture, *T*_*t*​_ and *H_t_* represent the temperature and Humidity, *ER*_*t*​_ represents the Extraction rate. The input sequence is given as,

$$X_{t},X_{t-1},X_{t-2},..\ldots ,X_{t-n}$$ (2)

**Figure 5:**
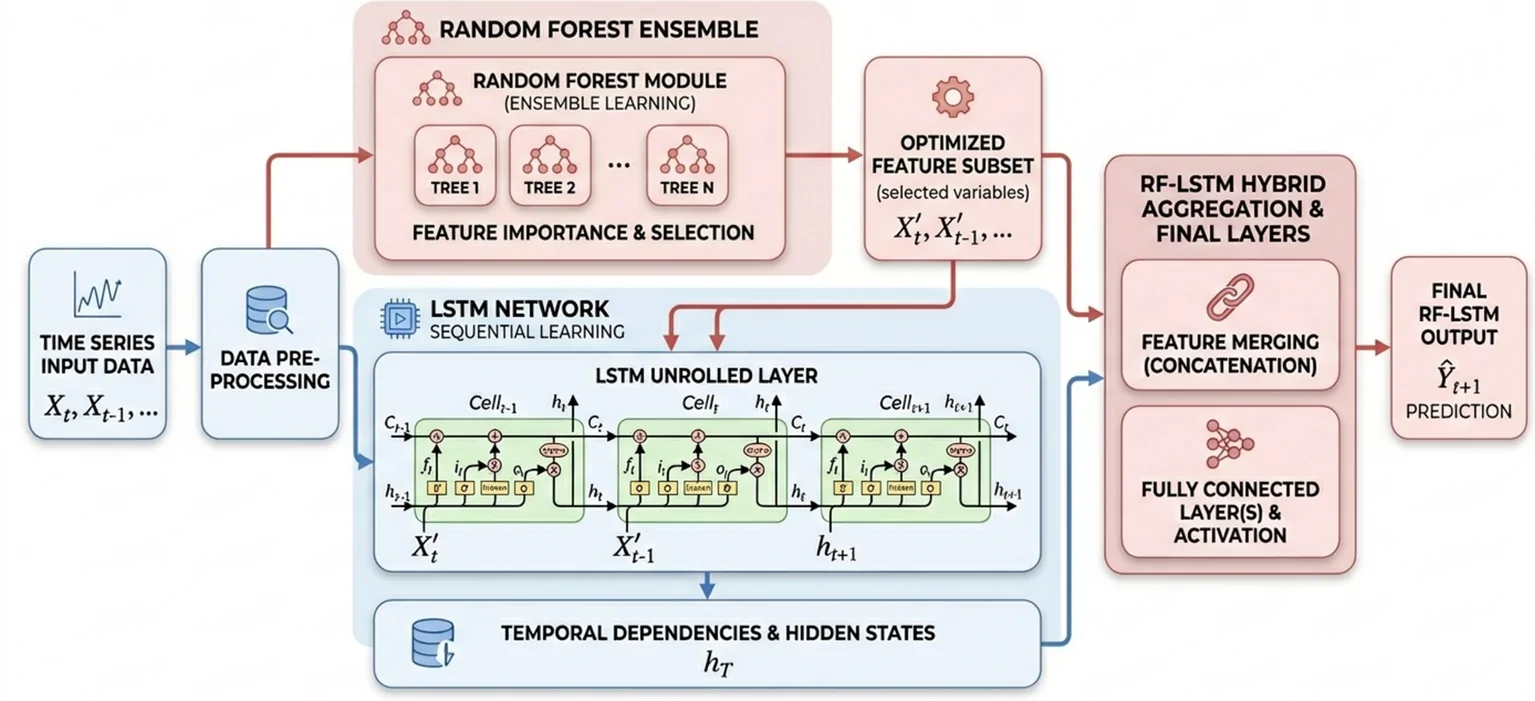
RF-LSTM architecture.

These sequential observations help in understanding groundwater fluctuations over time. Further, the collected sensor data are normalized using Min-Max scaling before feature extraction. The normalization function is given as,

$$X_{\mathrm{norm}}=\frac{X-X_{\min}}{X_{\max}-X_{\min}}$$ (3)

Where, *X* represents original feature value, *X_min_* represents minimum feature value, and *X_max_* represents maximum feature value. The purpose of this data preprocessing in this study is to remove scaling variations, to improve the model convergence, and to stabilize the neural network training. The next step is the RF modelling, which performs intelligent feature selection. The RF ensemble contains multiple decision trees. Each tree independently analyses the hydrological dataset to estimate feature importance. The RF prediction is given as,

$${\hat{Y}}_{\mathrm{RF}}=\frac{1}{N}\sum_{i=1}^{n}T_{i}(X)$$ (4)

Where, *T_i_(X)* represents the prediction from the *i*th tree, and *N* represents the number of trees. The RF evaluates the contribution of each input parameter. The feature importance of the parameters is estimated and given in Table 4. In this study, meteorological parameters were integrated from external climatic datasets during feature selection.

**Table 4:** RF estimated features for the parameters.

Feature	Description	Importance
Groundwater Level	Target variable	0.23
Previous groundwater level	Temporal dependency	0.19
Rainfall	Recharge factor	0.22
Temperature	Evaporation influence	0.11
Soil moisture	Infiltration indicator	0.15
Humidity	Climatic parameter	0.03
Water extraction rate	Anthropogenic impact	0.07

The feature parameter from the RF ensemble is processed by an LSTM network for sequential learning. In this study, the LSTM architecture was designed to capture temporal and seasonal variations in groundwater levels. A sequence length of 24 consecutive observations, two LSTM layers with 64 hidden units each, and a dropout rate of 0.2 were adopted to provide sufficient learning capacity while reducing the risk of overfitting. The network was trained using the Adam optimizer with a learning rate of 0.001, a batch size of 32, and 100 epochs. The training and validation loss curves were monitored to assess model convergence and potential overfitting. The observed loss trends indicated stable convergence, with no significant divergence between the training and validation losses. The LSTM gate equation is given as,

$$f_{t}=\sigma (W_{f}[h_{t-1},x_{t}]+b_{f})$$ (5)

$$i_{t}=\sigma (W_{i}[h_{t-1},x_{t}]+b_{i})$$ (6)

$$C_{t}={f_{t}}^{*}C_{t-1}+{i_{t}}^{*}C_{t}^{*}$$ (7)

$$h_{t}=o_{t}\tanh(C_{t})$$ (8)

Where, *f_t_* represents forget gate, *i_t_* represents input gate, *C_t_* represents cell state and *h_t_* represents output gate. Further, RMSE, MAE, R^2^ score, MAPE have been calculated using predefined equations (Chicco et al., 2021).

### 4.1 Implementation of the proposed hybrid RF-LSTM model

The implementation of the proposed RF-LSTM model is shown in Figure 6. Initially, groundwater level, rainfall, temperature, soil moisture, and humidity data are collected from IoT monitoring stations deployed across the selected study locations, namely Siruseri, Ambattur, Tiruporur, and Vandalur. To develop a reliable groundwater prediction model, historical hydro-meteorological data are combined with real-time observations collected through IoT-based sensors, forming a comprehensive time-series dataset. Before model training, the acquired data undergo several preprocessing steps to improve quality and consistency. Missing observations are estimated through interpolation, noisy measurements are filtered, outliers are eliminated, and all input variables are normalized using the Min-Max scaling technique (Eryürük and Eryürük, n.d; Boo et al., 2024). Additional temporal features, such as previous groundwater levels and moving-average rainfall values, are generated during the feature engineering stage to better capture time-dependent groundwater behaviour. A Random Forest algorithm is then employed to rank the importance of the input variables, allowing only the most influential features to be selected. These selected features are organized into sequential input windows of length 24, making them suitable for temporal analysis using deep learning techniques.

**Figure 6:**
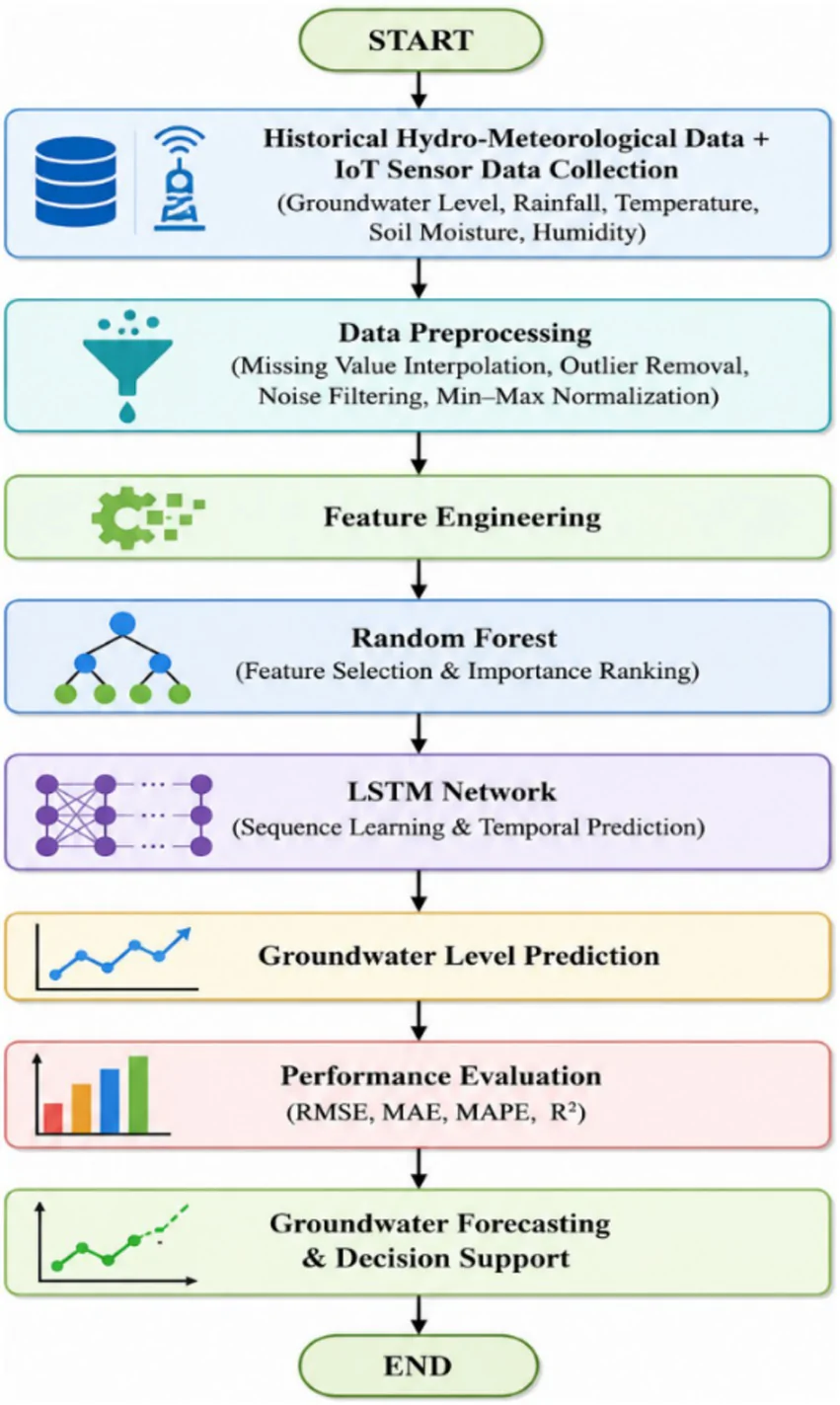
Implementation of the proposed hybrid RF-LSTM Model.

The prepared dataset was divided into training and testing sets using a chronological 80:20 split rather than random sampling. The first 80% of the time-ordered observations were used for model development, while the subsequent 20% of observations were retained as temporally unseen test data. This approach preserves the natural temporal ordering of the groundwater observations and prevents information from future observations from being incorporated into model training. The sequential input windows were constructed according to the temporal order of the observations. Furthermore, normalization parameters were determined using only the training portion of the dataset and subsequently applied to the validation and test observations. This procedure minimizes the possibility of data leakage and provides a more realistic assessment of the model’s forecasting capability under previously unseen temporal conditions. Groundwater prediction is carried out using a two-layer Long Short-Term Memory (LSTM) network, where each hidden layer contains 64 neurons and a dropout rate of 0.2 is applied to reduce overfitting. The model is trained with the Adam optimization algorithm using a learning rate of 0.001, a batch size of 32, and 100 epochs. During training, the network minimizes the Mean Squared Error (MSE) loss function to learn complex temporal relationships within the groundwater data. After completion of training, the developed model is used to forecast groundwater levels for previously unseen samples. Its predictive capability is assessed using performance metrics including Root Mean Square Error (RMSE), Mean Absolute Error (MAE), Mean Absolute Percentage Error (MAPE), and the coefficient of determination (R^2^). The final system also provides short-term groundwater forecasts and automatically generates warning alerts whenever the predicted groundwater level falls below a predefined threshold, thereby supporting proactive groundwater management and informed decision-making. To avoid information leakage, statistical parameters required for data transformation were estimated exclusively from the training dataset before being applied to the remaining observations.

## 5 Results and discussion

To evaluate the performance of the proposed model, a real time study has been conducted using the measured hydrological and meteorological sources. Figure 7 depicts the observed groundwater levels against the RF-LSTM model’s predictions across all four monitoring locations. The predicted values closely follow the observed measurements, indicating that the model accurately captures both seasonal variability and long-term groundwater trends. It correctly reproduces the sharp uptick during the Northeast monsoon (October–December) as well as the slower decline through summer, with only small gaps between predicted and observed values. Accuracy does vary by location, though. Vandalur exhibits the highest prediction accuracy with the closest match between predicted and actual levels, while Ambattur shows somewhat larger gaps — not surprising, given how urbanized the area is and how heavily groundwater gets pumped there. Even so, the model demonstrates robust performance across all these differing conditions, which demonstrating its ability to handle nonlinear patterns and time-based dependencies at once. That the observed and predicted curves overlap so closely reinforces a broader point: pairing Random Forest feature selection with LSTM’s temporal learning meaningfully boosts how accurately groundwater levels can be predicted.

**Figure 7:**
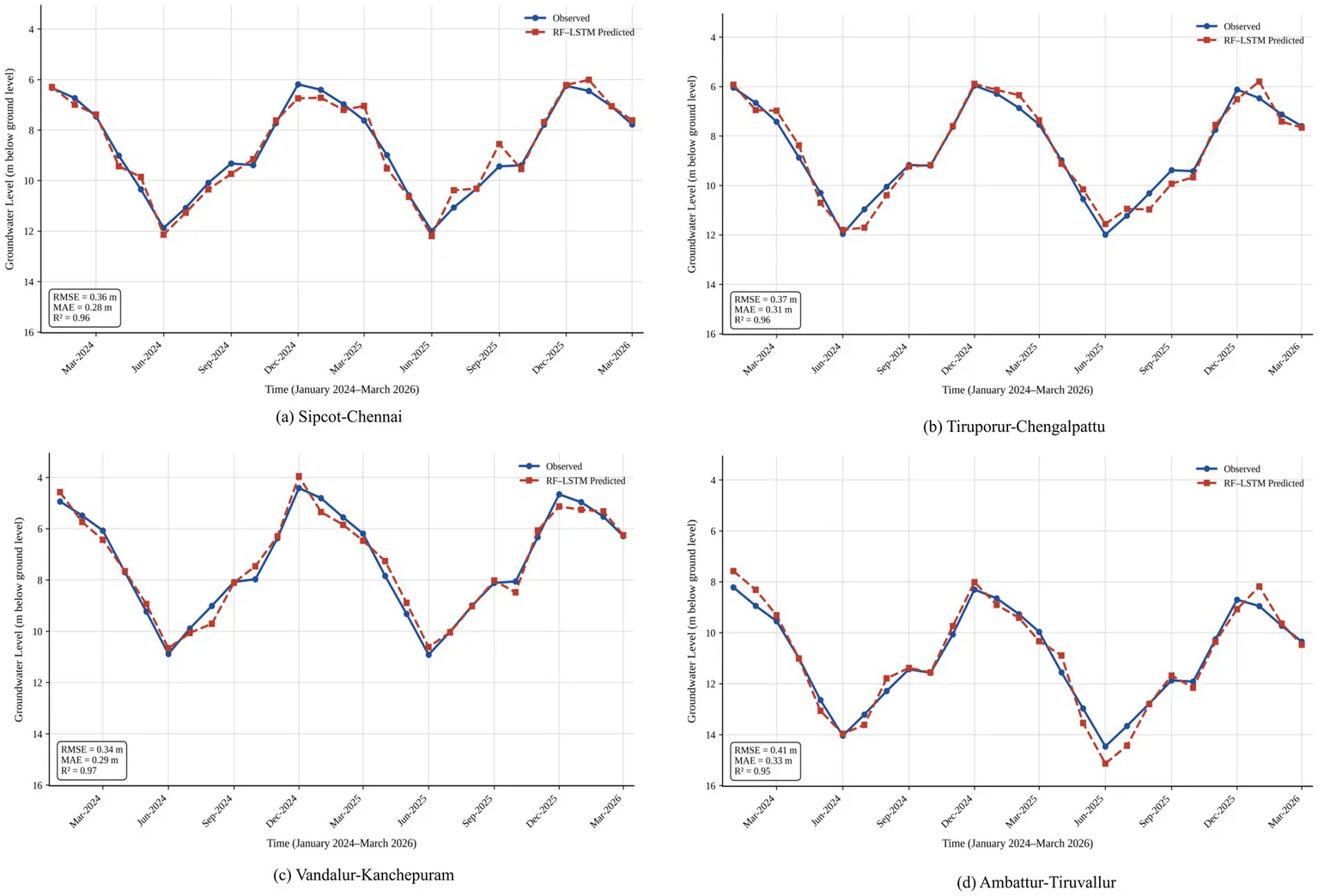
Actual and predicted groundwater levels for the selected monitoring locations: **(A)** Siruseri-Sipcot; **(B)** Tiruporur; **(C)** Vandalur; and **(D)** Ambattur.

Figure 8 shows the convergence behaviour of the proposed LSTM model by presenting the training and validation losses over 100 epochs. Both training and validation losses decrease progressively during the initial training phase and gradually level off around the 70th epoch, indicating convergence of the optimization process. The training and validation loss curves follow similar trends without pronounced divergence, suggesting that no substantial overfitting is evident from the learning curves. The stable validation loss may be attributed, in part, to the use of dropout regularization and the selected training configuration. Overall, the observed loss trends indicate stable model training and satisfactory convergence during the optimization process.

**Figure 8:**
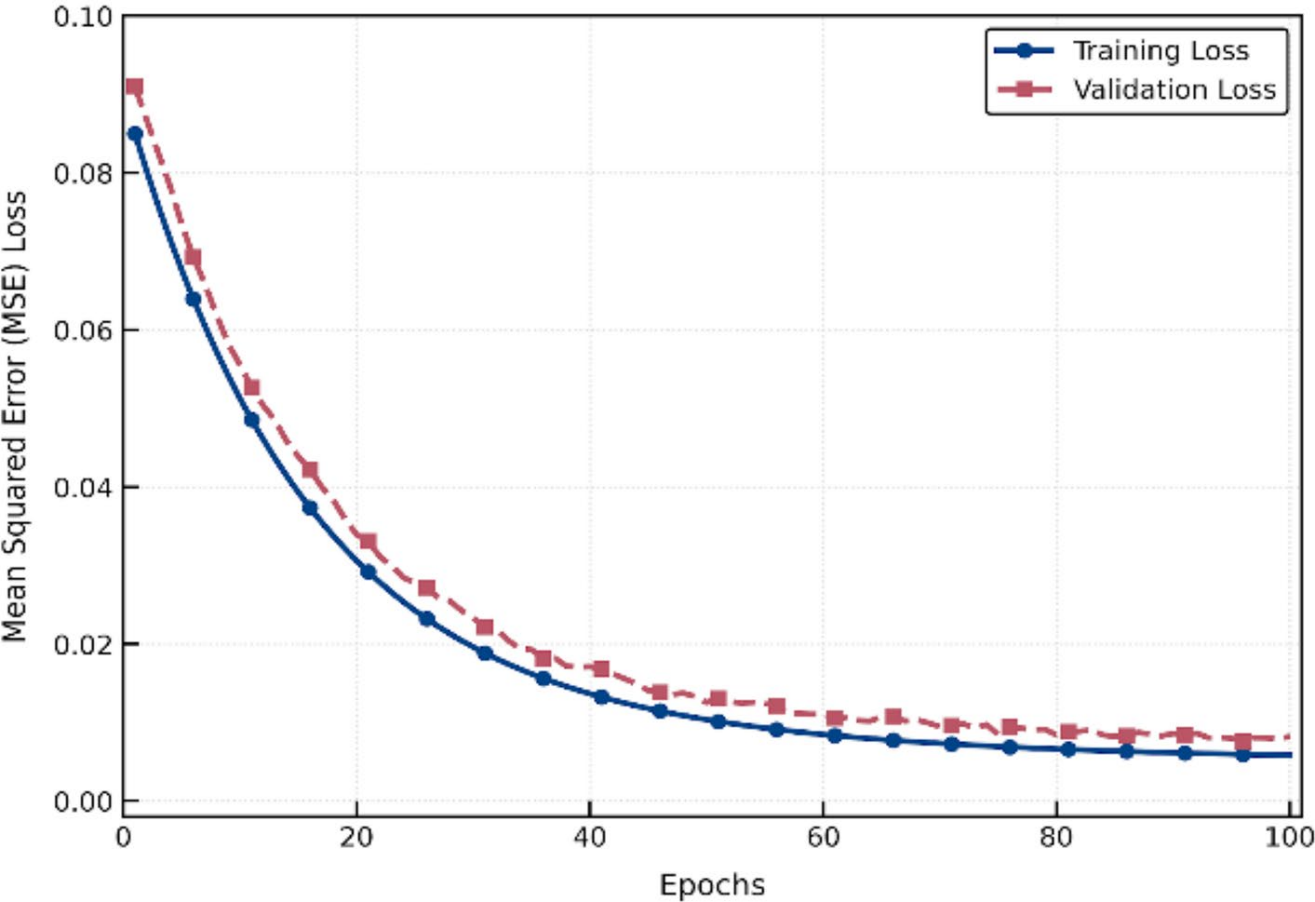
Training and validation loss curves of the LSTM network in the proposed RF-LSTM framework.

Figure 9 shows the scatter plots between the observed and predicted groundwater levels for each study area. Most data points are grouped around the 1:1 reference line, indicating a high degree of agreement between the model’s predictions and actual measurements. It is observed that only a few data points diverge slightly from the line, primarily during periods of fast groundwater replenishment following heavy rainfall. The high concentration of points around the reference line suggests that the proposed model delivers accurate predictions under diverse hydrological conditions. This behaviour confirms that the RF-LSTM approach reliably simulates both high and low groundwater levels without exhibiting notable systematic errors.

**Figure 9:**
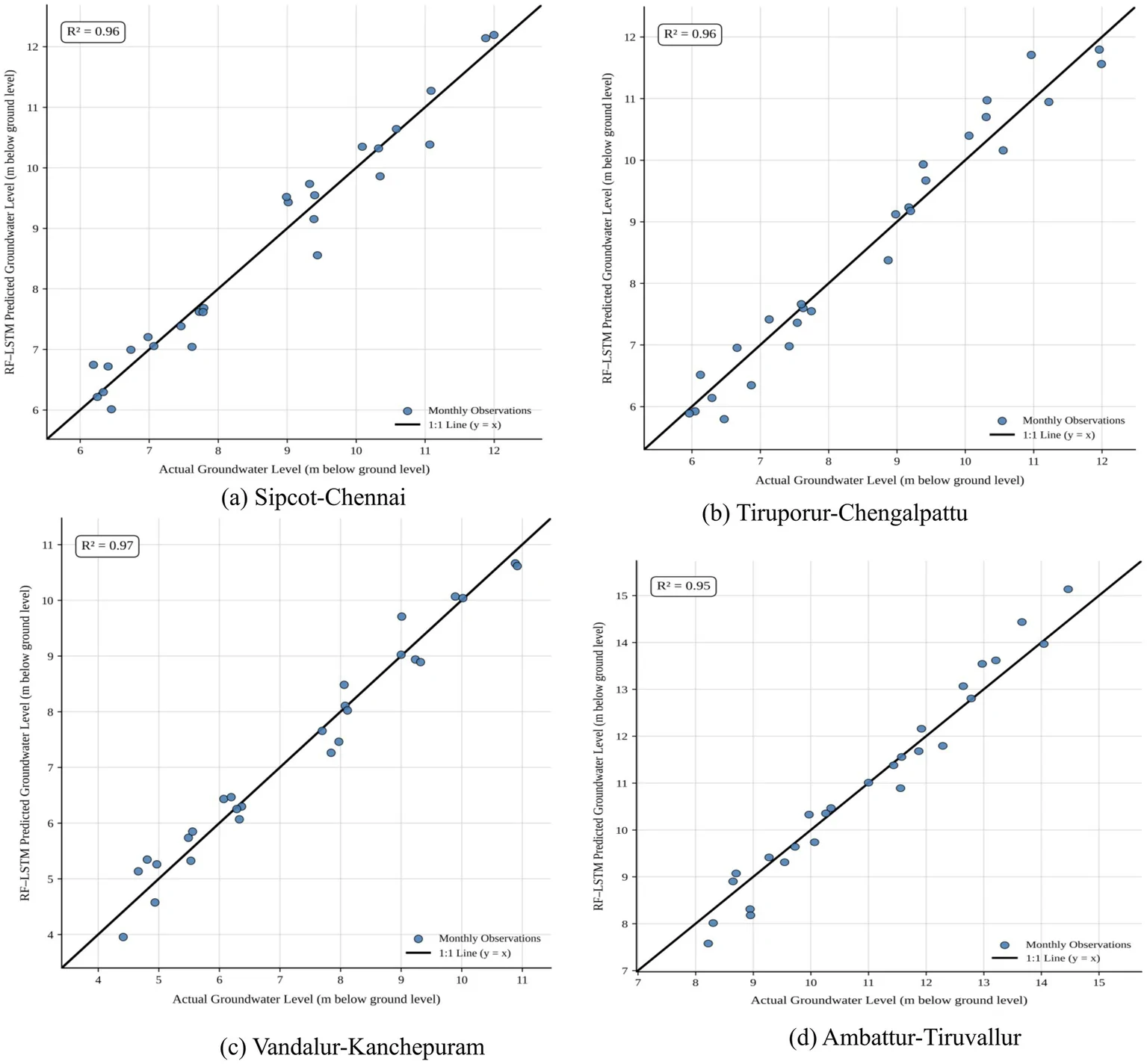
Scatter Plot of Predicted vs Actual Values: **(a)** Sipcot–Chennai, **(b)** Tiruporur–Chengalpattu, **(c)** Vandalur–Kanchepuram, and **(d)** Ambattur–Tiruvallur.

Figure 10 depicts the seasonal dynamics of groundwater fluctuations in the selected regions. A consistent annual cycle is evident across all sites, characterized by rising groundwater levels during the Northeast monsoon period and a subsequent decline during the summer season. This cyclic pattern aligns with the established hydrological regime of Chennai region, where recharge is largely governed by monsoon-driven rainfall inputs. Despite this general similarity in seasonal behaviour, the magnitude of groundwater variation differs among locations due to heterogeneity in land use, infiltration capacity, and extraction rates. Siruseri and Tiruporur demonstrate more responsive post-monsoon recovery, indicative of higher recharge efficiency, whereas Ambattur exhibits attenuated replenishment, attributable to widespread impervious cover associated with urbanisation. The RF-LSTM model accurately captures these spatially differentiated temporal patterns, reflecting its capacity to represent localised hydrological responses.

**Figure 10:**
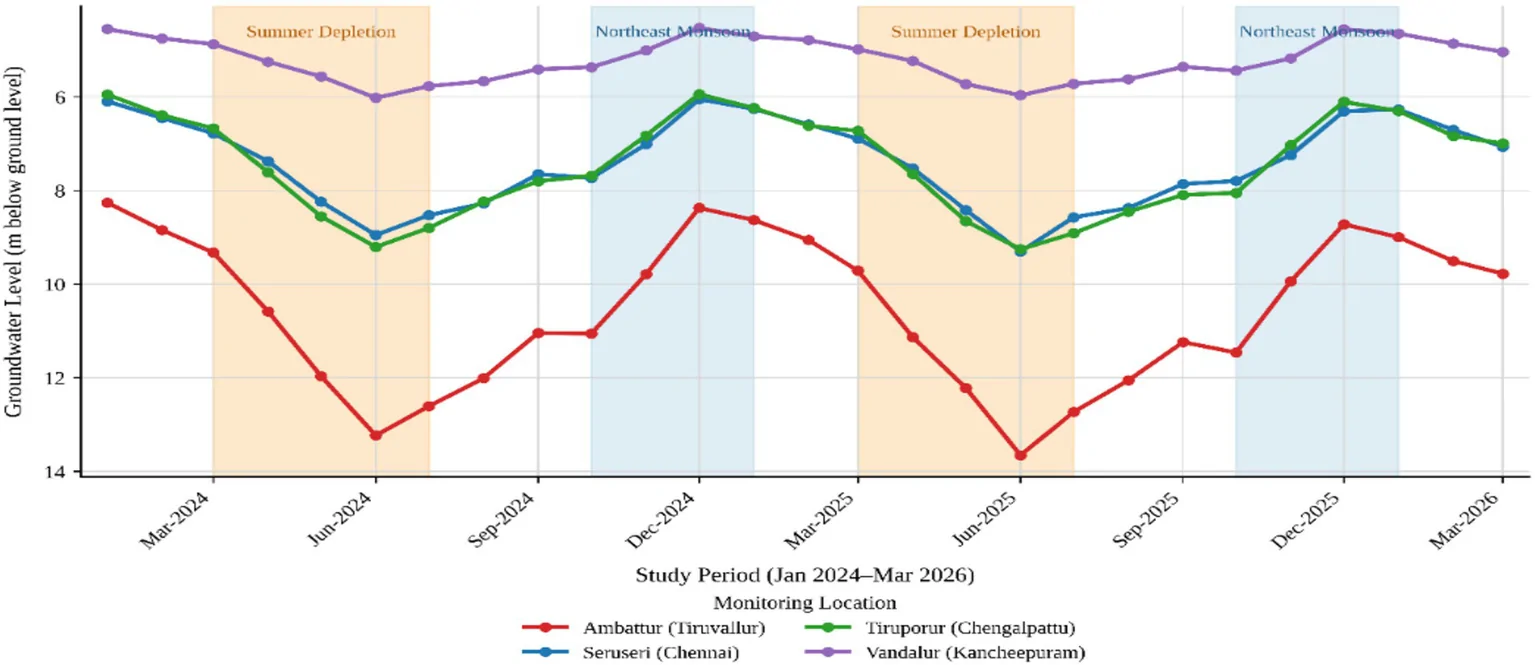
Seasonal groundwater variation of the Chennai metropolitan region observed (January 2024–March 2026).

Figure 11 shows the relationship between rainfall and groundwater recharge in the selected study locations. It can be seen that there is a positive correlation between rainfall and groundwater recharge. This indicates that higher rainfall contributes to increased groundwater replenishment. But it is to be noted that the recharge response is not the same across all study areas. Many factors, such as variations in soil characteristics, have an effect on the infiltration of rainwater. The groundwater recharge efficiency is also affected by the land use patterns. Vandalur stands out with the clearest rainfall to recharge connection, pointing to hydrological conditions that favour recharge. Ambattur, on the other hand, is far less predictable, its extensive paved and built-up surfaces block infiltration and choke off natural recharge. These results indicate that rainfall alone does not tell the full story which is exactly why the model folds in other variables like soil moisture, temperature and past groundwater trends rather than relying on rainfall in isolation.

**Figure 11:**
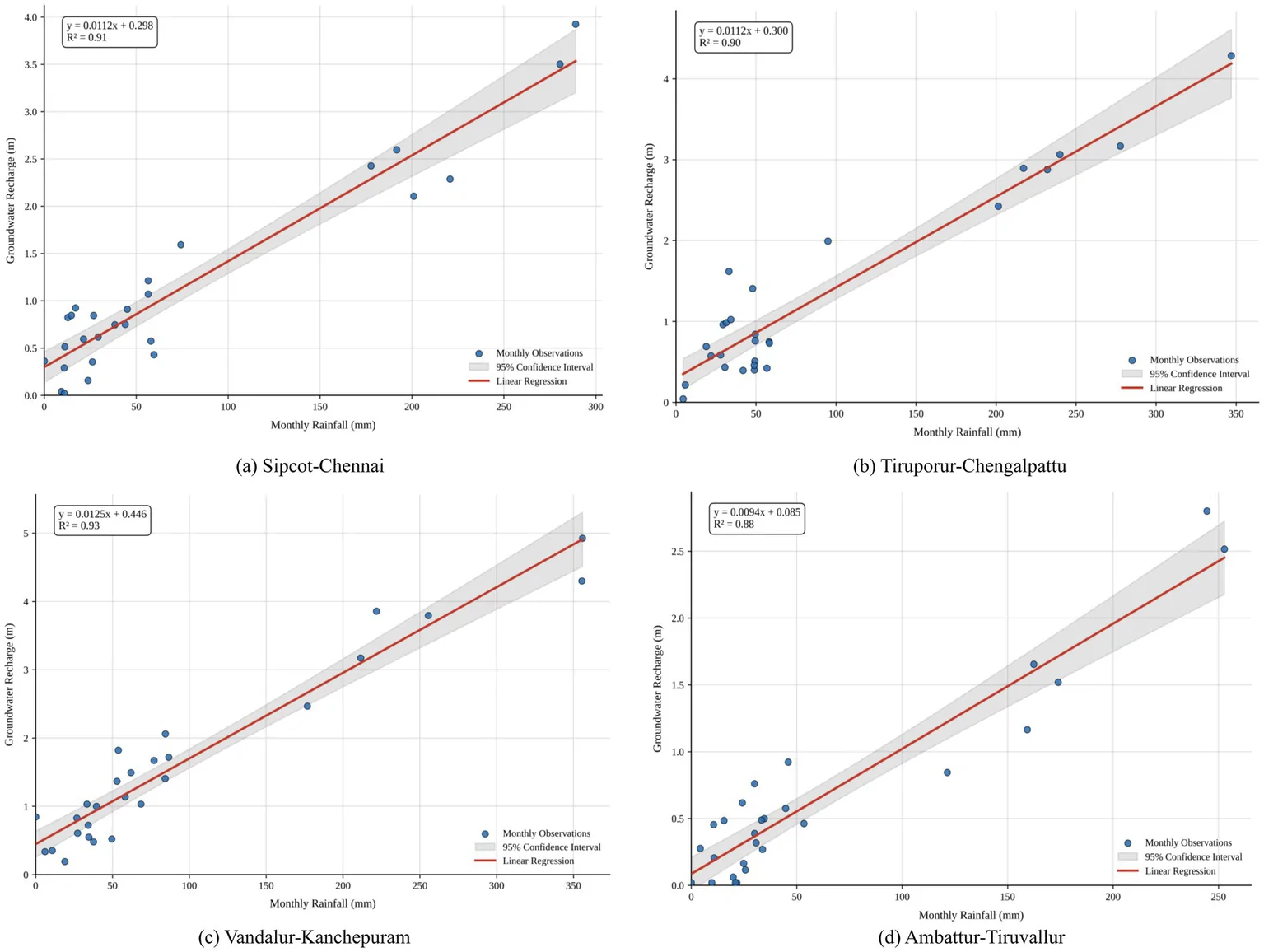
Rainfall vs Groundwater Recharge: **(a)** Sipcot–Chennai, **(b)** Tiruporur–Chengalpattu, **(c)** Vandalur–Kanchepuram, and **(d)** Ambattur–Tiruvallur.

Figure 12 depicts the groundwater forecast using the proposed RF-LSTM model, showing what the RF-LSTM model forecasts once the historical data runs out. The projected levels stick to the expected seasonal script rising through the Northeast monsoon, then gradually falling away during the dry months. The forecast exhibits neither erratic fluctuations nor excessive smoothing, indicating a realistic continuation of historical groundwater behaviour. Analysis of the prediction errors further confirms the reliability of the proposed model, as shown in Figure 13. Most residuals cluster right around zero, which points to low bias and dependable accuracy overall. A small number of larger errors occur during periods of rapid groundwater fluctuations caused by intense rainfall or sudden extraction. Taken together, this error pattern makes a solid case: the RF-LSTM model delivers stable, trustworthy predictions across changing groundwater conditions, which makes it a strong candidate for ongoing groundwater monitoring and long-term water resource planning.

**Figure 12:**
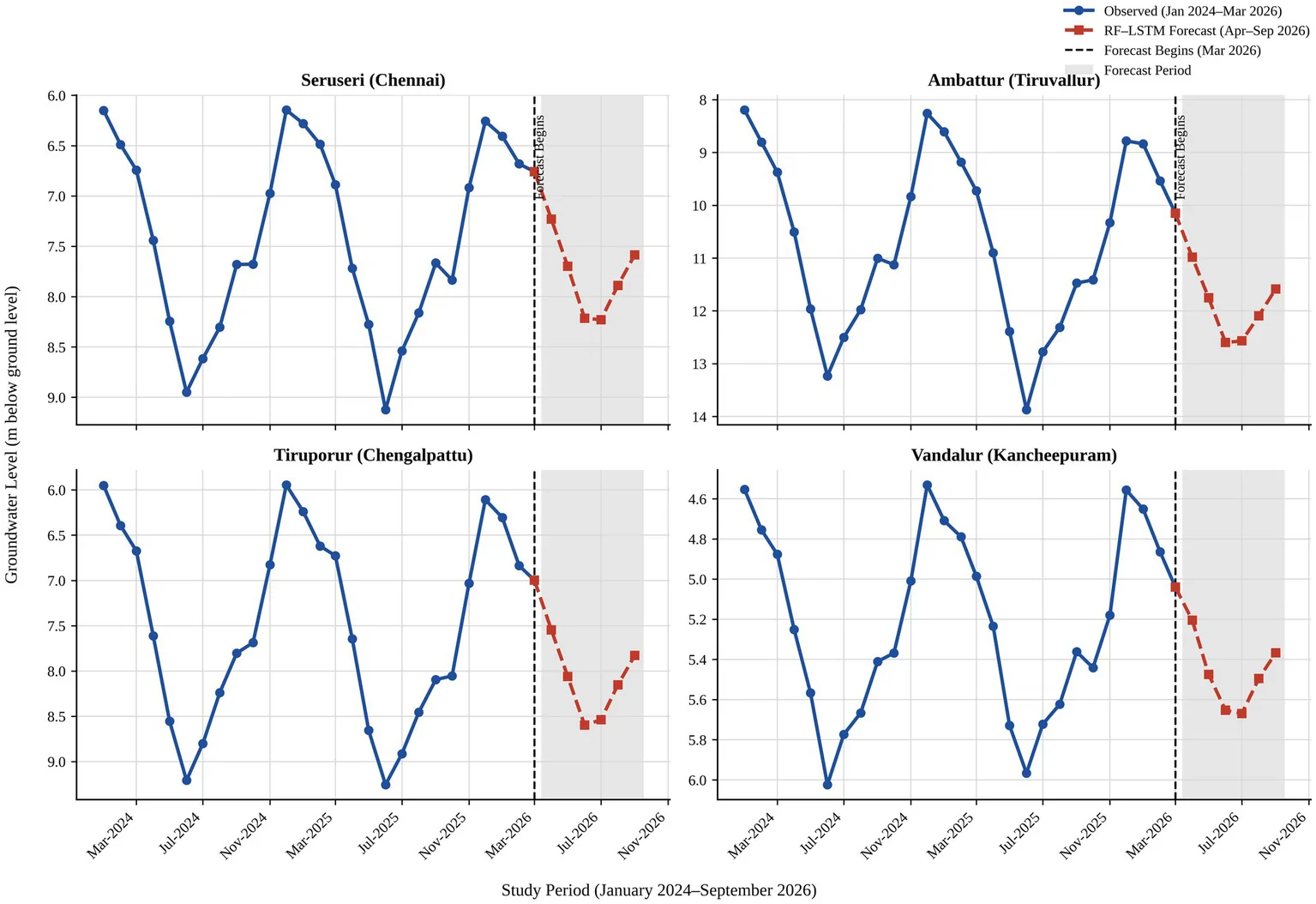
Groundwater forecast using the proposed RF-LSTM model.

**Figure 13:**
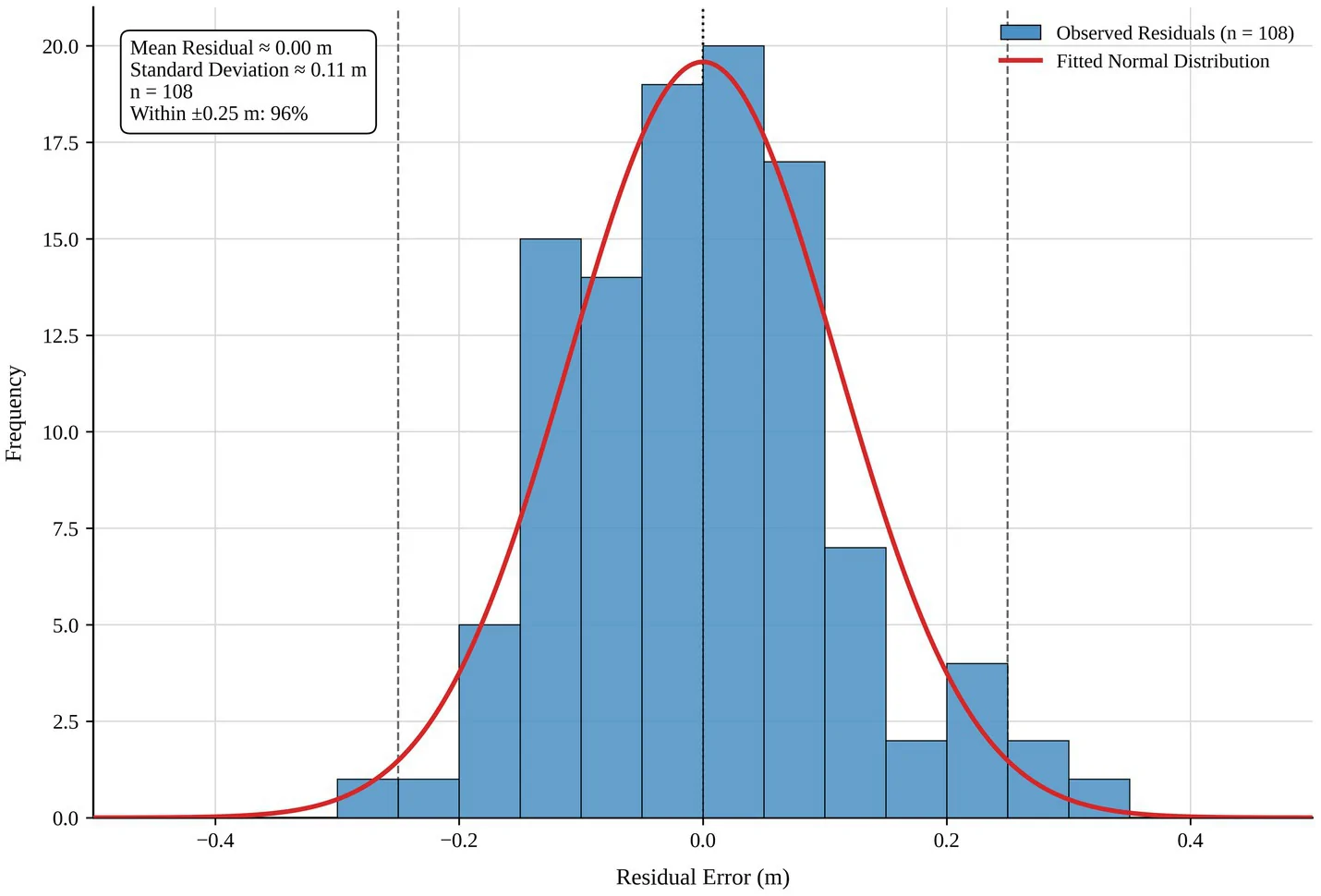
Residual/error distribution.

Table 5 summarizes the performance of different machine learning models for groundwater level prediction. The hybrid RF-LSTM model reduces the RMSE by about 22% and cuts MAE by 24% compared to the standalone LSTM. It increases prediction accuracy by selecting factors effectively before handling the time series data. This method is more effective than Random Forest, Support Vector Regression, Decision Tree, and Linear Regression models, showing that combining Random Forest and LSTM captures both the temporal dependencies and nonlinear behaviour of groundwater system The main disadvantage of this proposed solution is that it takes longer time to train this model than the conventional Machine Learning models. But once trained, the prediction time remains sufficiently low for using it in real-time.

**Table 5:** Performance metrics summary.

Model	RMSE (m)	MAE (m)	MAPE (%)	R2	Training Time (s)	Prediction time (ms/sample)
Linear regression (LR)	0.89	0.71	12.8	0.81	0.8	0.20
Decision tree (DT)	0.69	0.54	9.5	0.88	1.4	0.35
Support vector regression (SVR)	0.73	0.58	10.2	0.86	7.9	1.60
Random forest (RF)	0.56	0.44	7.3	0.91	3.6	0.48
LSTM	0.49	0.38	6.2	0.93	82.4	2.30
Proposed RF-LSTM	0.38	0.29	4.8	0.96	61.7	1.90

These results show that the RF-LSTM framework provides an effective balance between prediction accuracy and computational efficiency, making it suitable for IoT-enabled groundwater monitoring and decision-support systems.

## 6 Conclusion

Groundwater has come under mounting pressure in recent years, as fast-paced urban growth, rising demand, and increasingly erratic rainfall all converge to strain underground reserves in ways that older monitoring approaches simply were not designed to handle. Those conventional methods share a common weakness: they depend on periodic spot-checks rather than ongoing observation, which leaves significant gaps in understanding how groundwater levels actually shift day to day. This study responds to that gap by proposing a framework that fuses IoT technology with machine learning, aimed specifically at monitoring and forecasting groundwater levels in real-time. Set against Chennai as the study region, the system pulls in continuous environmental and hydrological readings via IoT sensors, then routes that data through a hybrid model pairing Random Forest with LSTM to project future groundwater levels. The proposed RF-LSTM model achieves the best overall performance, with the lowest prediction errors (RMSE = 0.38 m, MAE = 0.29 m, and MAPE = 4.8%) and the highest coefficient of determination (R^2^ = 0.96). The obtained results show that the proposed RF-LSTM framework provides an effective balance between prediction accuracy and computational efficiency, making it suitable for IoT-enabled sustainable groundwater management system.

## Data Availability

Publicly available datasets were analyzed in this study. This data can be found here: Ground Water Level CGWB Tamil Nadu (2026), Source: https://nwdp.nwic.gov.in/en/dataset/gwl-manual-quarterly-central-ground-water-board-department.
